# m^6^A: An Emerging Role in Programmed Cell Death

**DOI:** 10.3389/fcell.2022.817112

**Published:** 2022-01-24

**Authors:** Fajuan Tang, Lin Chen, Hu Gao, Dongqiong Xiao, Xihong Li

**Affiliations:** ^1^ Department of Emergency, West China Second University Hospital, Sichuan University, Chengdu, China; ^2^ Key Laboratory of Birth Defects and Related Diseases of Women and Children, Ministry of Education, Sichuan University, Chengdu, China

**Keywords:** m^6^A, autophagy, ferroptosis, pyroptosis, apoptosis, necroptosis

## Abstract

Programmed cell death is an active extinction process, including autophagy, ferroptosis, pyroptosis, apoptosis, and necroptosis. m^6^A is a reversible RNA modification which undergoes methylation under the action of methylases (writers), and is demethylated under the action of demethylases (erasers). The RNA base site at which m^6^A is modified is recognized by specialized enzymes (readers) which regulate downstream RNA translation, decay, and stability. m^6^A affects many aspects of mRNA metabolism, and also plays an important role in promoting the maturation of miRNA, the translation and degradation of circRNA, and the stability of lncRNA. The regulatory factors including writers, erasers and readers promote or inhibit programmed cell death *via* up-regulating or down-regulating downstream targets in a m^6^A-dependent manner to participate in the process of disease. In this review, we summarize the functions of m^6^A with particular reference to its role in programmed cell death.

## Cell Death

Programmed cell death (PCD) is the active process of cell death, including autophagy, ferroptosis, pyroptosis, apoptosis, and necroptosis (Cabon et al., 2013). These forms of cell death have different and independent regulatory pathways (Supplementary Table S1) (Kaiser et al., 2011; Newton et al., 2014; Broz and Dixit, 2016; New and Thomas, 2019; Uzdensky, 2019; Capelletti et al., 2020; Qiu et al., 2020; Beroske et al., 2021; Bhardwaj et al., 2021; Tsuchiya, 2021; Yuan et al., 2021a). However, there are interconnections and crosstalk between these pathways (Bedoui et al., 2020; Wang and Kanneganti, 2021). For example, Bcl-2 is not only an inhibitor of apoptosis but also a fundamental regulator of autophagy (Mukhopadhyay et al., 2014). Bcl-2 inhibits autophagy induced by Beclin-1 by binding to the autophagy-related protein Beclin-1 to form a complex (Mukhopadhyay et al., 2014). Caspase-8 acts as a molecular switch and is activated in response to cell death signals to play a key role in apoptosis and necroptosis (Fritsch et al., 2019; Schwarzer et al., 2020). A study of lysosomal storage disease has shown that lysosomal dysfunction affects the accumulation of autophagosomes to induce autophagy-dependent ferroptosis (Pierzynowska et al., 2021). A previous study of the pathological mechanisms underlying renal tubular necrosis found that necroptosis initiated the cell death process *via* ferroptosis (Belavgeni et al., 2020). Interestingly, it was proved that these forms of cell death can be mediated by post-transcriptional regulation, including N^6^-methyladenosine (m^6^A) (Song et al., 2019).

## Overview of m^6^A

More than 100 different modifications have been identified in coding RNA and non-coding RNA (ncRNA) (Boccaletto et al., 2018). Methylation modification is one of the most common form of RNA modification, usually including N^1^-methyladenosine (m^1^A), 5-methylcytosine (m^5^C), 5-hydroxymethylcytosine (5hmC), m^6^A and 7-methylguanine (m^7^G) (Boccaletto and Bagiński, 2021). m^6^A was first discovered by Desrosiers et al. in the mRNA of liver cancer cells in 1974 and acted as the most prominent and abundant internal RNA modification in mammalian RNA (Desrosiers et al., 1974; Coker et al., 2019; Muthusamy, 2020). Adenosine can be chemically modified by adding methyl groups to the adenine bases in RNA. m^6^A occurs when the N^6^ position of adenosine is methylated (Oerum et al., 2021). This process is dynamically regulated by methylase and demethylase, and plays its functional role under the action of corresponding recognition enzymes (Fu et al., 2014).

## Enzymes Involved in m^6^A

**FIGURE 1:**
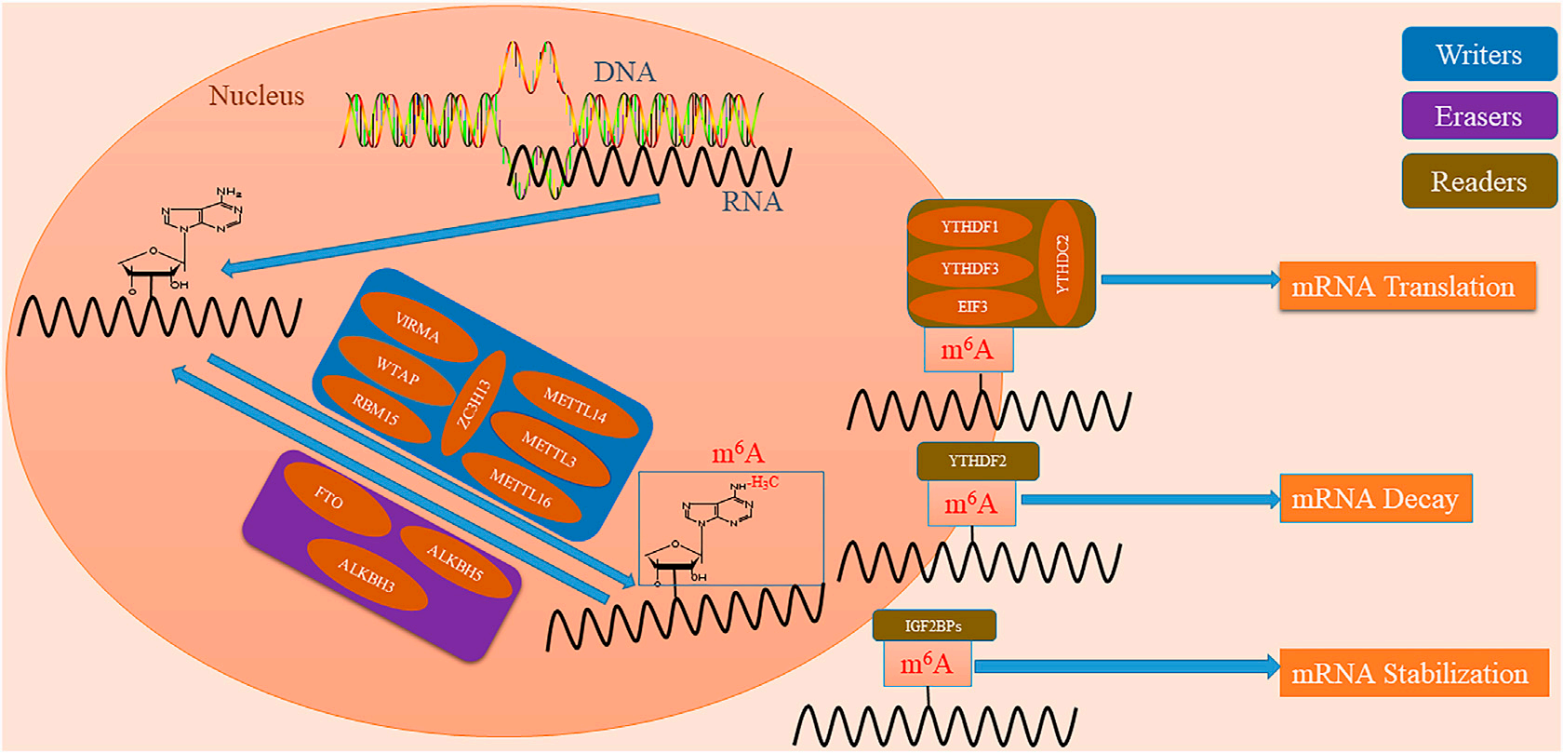
Mechanisms underlying the m6A modification. m^6^A modification is a reversible process. Writers represent methylases which include METTL3, METTL14, WTAP, VIRMA/KIAA1429, RBM15, ZC3H13, and METTL16, to make the N^6^ position of adenosine methylated. Erasers represent demethylases including FTO, ALKBH5 and ALKBH3, to make m^6^A demethylated. Readers represent specialized enzymes, including YTHDF1, YTHDF2, YTHDF3, YTHDC1, YTHDC2, IGF2BPs, and EIF3, which recognize the RNA base site at which m6A is modified, to regulate downstream mRNA translation, degradation, and stabilization.

Installation of m^6^A is a reversible process regulated by the methylases (writers) and demethylases (erasers) (Supplementary Table S2) (Figure 1) (Karthiya and Khandelia, 2020). For one thing, the writers form the methyltransferase complex (MTC) and catalyze the process for m^6^A (Bokar et al., 1994; Śledź and Jinek, 2016). Methyltransferase-like protein 3 (METTL3) is an S-adenosylmethionine (SAM) binding protein which catalyzes the transfer of methyl groups in SAM (Wang et al., 2016). Methyltransferase-like protein 14 (METTL14) is another active component of MTC and forms a stable complex with METTL3 (in a ratio of 1:1) to stabilize the structure of MTC (Wang et al., 2016). WT1 associated protein (WTAP) recruits METTL3 and METL14 to nuclear areas, and RNA binding motif protein 15 (RBM15) guides METTL3 and WTAP to RNA sites (Patil et al., 2016; Schöller et al., 2018). VIRMA/KIAA1429 recruits MTC and mediates the methylation of adenine bases near the 3′- untranslated region (UTR) (Yue et al., 2018). Zinc finger CCCH domain-containing protein 13 (ZC3H13) enhances its catalytic function by interacting with WTAP (Knuckles et al., 2018). Methyltransferase-like protein 16 (METTL16) is a newly discovered independent RNA methylase that catalyzes the installation of m^6^A (Warda et al., 2017). For another, m^6^A undergoes demethylation under the action of erasers (Jia et al., 2011). FTO that proves the reversibility of the methylation process is the first protein identified to catalyze the demethylation of m^6^A (Jia et al., 2011). ALKBH5 is the second demethylase identified to reverse m^6^A, catalyzing the demethylation of m^6^A in a Fe(II) and *α*-ketoglutarate-dependent manner (Aik et al., 2014). In addition, a recent study identified ALKBH3 as a new demethylase involved in this process with a similar mechanism (Ueda et al., 2017).

Interestingly, the RNA base site at which m^6^A is modified is recognized by specialized enzymes (readers) which regulate downstream mRNA translation, decay, and stability (Supplementary Table S2) (Figure 1) (Meyer et al., 2015; Huang et al., 2018; Shi et al., 2021). YTHDF1 recruits initiation factors to promote mRNA translation and protein synthesis (Wang et al., 2015). YTHDF2 recruits mRNA modified by the selective binding of m^6^A to the mRNA decay site to induce the degradation of transcripts (Wang et al., 2015). YTHDF3 has been shown to combine with YTHDF1 and YTHDF2 to enhance their function (Lasman et al., 2020). YTHDC1 promotes RNA splicing and export by recruiting mRNA splicing factors (Woodcock et al., 2020). YTHDC2 interacts with RNA helicase to improve the translation efficiency of target RNA (Kim and Siddiqui, 2021). IGF2BPs and EIF3 play important roles in improving the stability of mRNA and the efficiency of the translation initiation complex (Lee et al., 2016; Müller et al., 2019). Heterogenous nuclear ribonucleoproteins (HNRNPs) are a complex and functionally diverse family of RNA binding proteins (Low et al., 2021). HNRNPA2/B1 as a member of the HNRNPs family, is identified as a nuclear reader of m^6^A and regulates the alternative splicing of exons in a set of transcripts (Alarcón et al., 2015).

## The Function of m^6^A

m^6^A is one of the most prominent RNA modification for mRNA and ncRNA and plays a variety of biological functions by regulating these forms of RNA (Supplementary Table S3(Roignant and Soller, 2017; Dai et al., 2018; He et al., 2020; Zhang et al., 2020; Han et al., 2021).

### m^6^A in mRNA Metabolism

Transcription of mRNA is the first step which leads to the production of protein, and the post-transcriptional control of mRNA is regulated by multiple mechanisms (Ivanov and Anderson, 2013). m^6^A modification affects many aspects of mRNA metabolism, including processing, export, translation, and decay (Roignant and Soller, 2017; Dai et al., 2018). mRNA processing promotes mRNA maturation through 5′capping, 3′polyadenylation and splicing (Dai et al., 2018). FTO depletion or METTL3 overexpression is known to increases m^6^A levels and promote the splicing of arginine-rich splicing factor (SRSF) (Zhao et al., 2014). YTHDC1 promotes RNA splicing and export by recruiting SRSF (Woodcock et al., 2020). HNRNP may affect the abundance and alternative splicing of target mRNA in a manner regulated by the “m^6^A switch” (Alarcón et al., 2015).

The nuclear export and translation of mRNA are key steps in the regulation of gene expression; m^6^A modification is known to facilitate this process (Dai et al., 2018). The knockdown of METTL3 was shown to reduce mRNA export to the cytoplasm, while inhibition of ALKBH5 had the opposite effect (Song et al., 2019). EIF3 plays an important role in improving the efficiency of mRNA translation initiation complex; YTHDF1 and METTL3 can recruit EIF3 to promote the translation of m^6^A-modified mRNA (Wang et al., 2015; Lee et al., 2016). Interestingly, YTHDF3 promotes mRNA translation in an m^6^A-dependent manner by enhancing the function of YTHDF1 (Lasman et al., 2020).

RNA decay is characterized by deadenylation which regulated by m^6^A modification (Du et al., 2016). YTHDF2 directly interacts with the SH domain of CCR4-NOT transcription complex subunit 1 (CNOT1) to mediate mRNA deadenylation (Du et al., 2016). YTHDF2 has also been shown to recruit mRNA modified by the m^6^A to mRNA decay sites to induce decay (Wang et al., 2015).

### m^6^A in ncRNA

ncRNA includes microRNA (miRNA), long non-coding RNA (lncRNA), and circular RNA (circRNA). m^6^A modification regulates cell proliferation, apoptosis, and the cell cycle by promoting the maturation of miRNA, the translation and degradation of circRNA, and the stability of lncRNA (He et al., 2020; Zhang et al., 2020; Han et al., 2021). Cigarette smoke condensate (CSC) mediates METTL3 to promote the maturation of miRNA-25-3 to enhance pancreatic ductal adenocarcinoma (Zhang et al., 2019). In addition, METTL3 can enhance the binding ability of pri-miRNA-221/222 and DGCR8 to promote bladder cancer (Han et al., 2019). METTL14 promotes the maturation of pri-miRNA-126 to inhibit the invasion of hepatocellular carcinoma (Ma et al., 2017). Some circRNAs have potential protein coding capabilities, and the process can be driven by m^6^A (Zhang et al., 2020). This process is initiated by YTHDF3, enhanced by METTL3/14, and inhibited by FTO (Zhang et al., 2020). Surprisingly, m^6^A modification can also promote circRNA degradation (Zhang et al., 2020). HRSP12 is an adaptor protein that can connect YTHDF2 and RNase P/MRP (endoribonuclease). circRNA is cleaved by endoribonuclease through the YTHDF2-HRSP12-RNase P/MRP axis; m^6^A promotes this process (Dong et al., 2019). m^6^A modification is known to affect the carcinogenic role of MALAT1 by promoting the binding ability with HNRNP C (Yang et al., 2013). Moreover, m^6^A modification can increase the stability of FAM225A and up-regulate its level to promote the proliferation and invasion of nasopharyngeal carcinoma (Zheng et al., 2019). Silencing METTL3 will reduce the m^6^A level and stability of total RNA of FAM225A (Zheng et al., 2019).

## The Role of m^6^A in PCD

**FIGURE 2:**
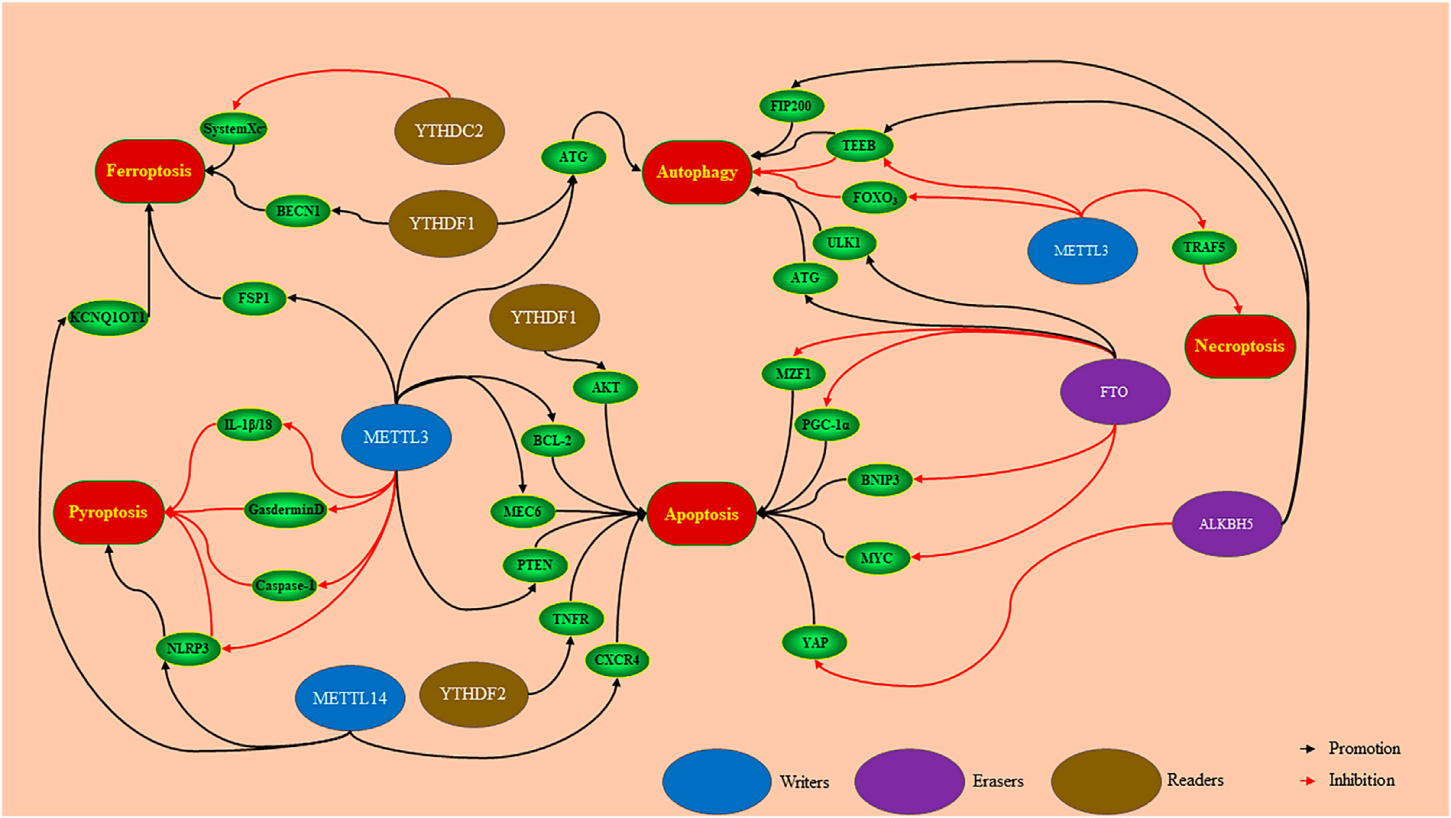
Writers, erasers and readers mediates downstream targets to regulate PCD in a m6A-dependent manner. METTL3 promotes ferroptosis and apoptosis, and inhibits necroptosis, *via* regulating downstream targets. According to the different functions of downstream targets, METTL3 can inhibit or promote pyroptosis and autophagy. METTL14 regulates downstream targets to promote ferroptosis, pyroptosis, and apoptosis. FTO and ALKBH5 mediate downstream targets to promote autophagy and apoptosis. The YTH family plays a role in promoting ferroptosis, autophagy, and apoptosis according to mediate different target proteins.

PCD works a key role in the pathogenesis of cancer, neurodegenerative diseases, and inflammation (Florean et al., 2019; Heckmann et al., 2019). m^6^A is controlled by regulatory factors (writers and erasers) and recognition factors (readers) to mediate downstream targets to regulate PCD (Figure 2) (Song et al., 2019; Liu et al., 2020; Wang et al., 2020a; Tsuruta et al., 2020).

### m^6^A and Autophagy

Autophagy is a basic pathway of cellular degradation with a lysosomal-assisted degradation mechanism, which is characterized by the formation of autophagosomes (Frankel et al., 2017). Autophagy-related genes (ATG), uncoordinated 51-like kinase 1 (ULK1) and transcription factor EB (TFEB) are important regulators of autophagy (Bhardwaj et al., 2021). m^6^A modification is known to exert effects by strictly regulating these regulators in different disease backgrounds, which include direct inhibition, the formation of autophagosomes, initiation, and enhancement on autophagy (Song et al., 2019; Liu et al., 2020; Wang et al., 2020a). TFEB is a key transcription factor that regulates the function of lysosome and expression of ATG (Corà et al., 2021). The expression of METTL3 is known to increase in mouse models of ischemic heart disease and inhibits autophagy by down-regulating TFEB; but ALKBH5 can make the opposite effect by up-regulating TFEB (Song et al., 2019). FTO up-regulates ULK1 that is a positive regulator of autophagy to initiate autophagy in a m^6^A-dependent manner (Jin et al., 2018). FTO is identified to increase the expression of ATG5 and ATG7, and promote the autophagosome formation to promote both autophagy and adipogenesis (Jin et al., 2018; Wang et al., 2020a). In addition, the overexpression of METTL3 is proved to increase the level of m^6^A modification of ATG5 while the knockdown of ATG5 reduces the autophagy induced by METTL3, indicating that ATG5 is a key target to induce autophagy (Chen et al., 2021). YTHDF1 promotes the translation of the ATG gene by combining with m^6^A-modified ATG2 and ATG14 mRNA, thereby promoting autophagy in human hepatocellular carcinoma (Li et al., 2021).

Forkhead box O_3_ (FOXO_3_) is a key transcription factor in a variety of carcinogenic pathways (Lin et al., 2020). METTL3 affects FOXO_3_ RNA stability in a YTHDF1-dependent manner to inhibit the expression of FOXO_3_ to inhibit autophagy; The reduced expression of METTL3 increases the number of autophagosomes to activate autophagy in liver cancer cells (Lin et al., 2020). FIP200 is an essential autophagy gene, and ALKBH5 enhances autophagy by mediating the demethylation of FIP200 mRNA (Li et al., 2020a). Surprisingly, one study found that reduced levels of FTO can weaken the activation of the mTORC1 pathway to enhance autophagy (Gulati et al., 2013). FTO regulates mTOR signaling by mediating m^6^A demethylation and activates autophagy to promote cell proliferation in melanoma (Yang et al., 2019). In short, m^6^A modification has different roles in the regulation of autophagy in different disease backgrounds.

### m^6^A and Ferroptosis

Ferroptosis is a newly discovered type of programmed cell death that involves iron-dependent lipid peroxidation along with the loss of glutathione peroxidase 4 and dense mitochondrial membrane (Dixon and Stockwell, 2014). m^6^A modification, as a new form of post-transcriptional regulatory mechanism, is known to play an important role in ferroptosis (Shen et al., 2021; Zhuang et al., 2021). During the ferroptosis of liver fibrosis, the expression of METTL4 was shown to up-regulated; in addition, the level of m^6^A was shown to increase and was enhanced by ferroptosis inducers (Shen et al., 2021). Previous researchers investigated the mechanisms underlying the effect of doxorubicin on cardiotoxicity and found that doxorubicin induced the up-regulation of METTL14 expression and catalyzed the m^6^A modification of RNA KCNQ1OT1 to participate in the ferroptosis of cardio myocytes (Zhuang et al., 2021). Moreover, METTL3 acts as the target of miR-4443, and regulates the expression of FSP1 to mediate the ferroptosis of non-small cell lung cancer (Song et al., 2021). Autophagy may also represent a targeted pathway by which to regulate the sensitivity of cells to ferroptosis (Gryzik et al., 2021). BECN1 is a key protein that regulates autophagy and promotes ferroptosis by regulating the activity of the cysteine/glutamate antiporter (also referred to as system Xc-) (Kang et al., 2018). RNA-seq analysis has shown that m^6^A modification triggers the activation of autophagy by stabilizing BECN1 mRNA and by inducing ferroptosis; YTHDF1 can promote this process by recognizing the m^6^A binding site in the coding region of BECN1 (Shen et al., 2021). Studies have also found that YTHDC2 is a powerful endogenous inducer of ferroptosis and plays a predominant role in the treatment of lung adenocarcinoma by targeting the SLC3A2 subunit of system Xc^−^ (Ma et al., 2021).

### m^6^A and Pyroptosis

Pyroptosis is a cell death pathway that is characterized by the activation of various caspases including caspase-1 which is mediated by inflammasomes and causes cell perforation (Mulay, 2019). Studies have suggested that m^6^A plays a key role in the regulatory pathway responsible for pyroptosis in cells (Zha et al., 2020; Liu et al., 2021). NOD-like receptor protein 3 (NLRP3) is a key component of the inflammasome that causes pyroptosis and is known to promote pyroptosis and increase the levels of pro-inflammatory cytokines (Mulay, 2019). High glucose levels can induce the increased expression of pyroptosis-related proteins (Caspase-1, Gasdermin D, NLRP3, IL-1*β*, and IL-18) to lead to cell death (Zha et al., 2020). The overexpression of METTL3 reduces the expression of these proteins and reduces the extent of damage while the knockdown of METTL3 will aggravate cellular damage (Zha et al., 2020). Previous study investigated the manner by which interferon regulatory factor-1 (IRF-1) can promote the pyroptosis of macrophages in patients with acute coronary syndrome and detected increased levels of m^6^A and METTL3 in the macrophages (Guo et al., 2020). The overexpression of IRF-1 can induce an increase in the levels of m^6^A and METTL3 to promote acute coronary syndrome (Guo et al., 2020). A research on intervertebral disc degeneration found that METTL14 specifically induced an increase in the m^6^A modification of NLRP3 mRNA and increased the expression of NLRP3 protein (Yuan et al., 2021b).

### m^6^A and Apoptosis

Apoptosis is the spontaneous and orderly death of cells triggered by the endogenous mitochondrial pathway (BCL-2 pathway) and the exogenous death receptor pathway (Clavier et al., 2016). Recent studies have shown that apoptosis is regulated by m^6^A (Dominissini et al., 2012). More and more studies have shown that m^6^A plays a role in the occurrence of cancer by regulating apoptosis-related proteins to promote or inhibit apoptosis (Supplementary Table S4(Liu et al., 2018; Su et al., 2018; Niu et al., 2019; Tang et al., 2019; Zhuang et al., 2019). METTL3 is an important methylase for m6A modification, and it shows important performance in lung cancer, breast cancer, ovarian cancer, gastrointestinal cancer, and leukemia by regulating downstream targets BCL-2, MEC6, and PTEN to inhibit apoptosis (Vu et al., 2017; Wei et al., 2019; Wang et al., 2020b; Wang et al., 2020c; Bi et al., 2021). METTL3 is up-regulated in breast cancer cells (Wang et al., 2020b). The knockdown of METTL3 can reduce methylation levels, target Bcl-2, reduce proliferation, accelerate cell apoptosis, and inhibit tumor growth (Wang et al., 2020b). Similarly, METTL3 has been shown to mediate apoptosis by regulating Bcl-2 in lung cancer (Wei et al., 2019). In a mouse model of leukemia, the depletion of METTL3 was shown to induce cell differentiation and apoptosis *via* the same mechanism to prevent the progression of leukemia in mice (Vu et al., 2017). Moreover, METTL14 is elevated in breast cancer and regulates chemokine receptor 4 (CXCR4) to inhibit apoptosis (Sun et al., 2020). Interestingly, as an important demethylase, FTO promotes apoptosis and participates in the occurrence of cancer by regulating downstream targets BNIP3, MZF1, MYC, and PGC-1*α* (Liu et al., 2018; Su et al., 2018; Niu et al., 2019; Zhuang et al., 2019). BNIP3 is considered to be a pro-apoptotic member of the Bcl-2 apoptotic protein family (Gorbunova et al., 2020). In breast cancer, the expression of FTO is up-regulated while that of BNIP3 is down-regulated (Niu et al., 2019). Silencing FTO promotes an increase in the levels of BNIP3 mRNA and protein (Niu et al., 2019). FTO demethylates m^6^A in the 3′-UTR of BNIP3 and reduces its expression; this promotes the proliferation, colony formation, and metastasis of breast cancer cells (Niu et al., 2019). Another demethylase ALKBH5 plays a role in osteosarcoma by regulating YAP (Yuan et al., 2021c). In addition, the methylation recognition enzyme YTHDF1/2 has also received important attention in cancer, and studies have found that they affect the occurrence of liver cancer and leukemia by regulating AKT and TNFR (Bian et al., 2020; Li et al., 2020b).

### m^6^A and Necroptosis

Necroptosis is a lysed form of PCD that can cause inflammation (Negroni et al., 2020). Tumor necrosis factor (TNF), Toll-like receptor (TOLLR) family members, interferon, and other mediators, are the main factors that can lead to necroptosis (Shirjang et al., 2019). TRAF5 is a member of the TNF receptor-related factor family and can exert influence on cell survival, proliferation, differentiation, and death, by mediating a variety of downstream signaling pathways (Potter et al., 2007). Previous researchers investigated the mechanisms underlying resistance to oxaliplatin in colorectal cancer and found that the mRNA and protein levels of TRAF5 increased when METTL3 was knocked out; the overexpression of METTL3 had the opposite result (Lan et al., 2021). METTL3 is known to inhibit necroptosis by inhibiting the levels of TRAF5 m^6^A (Lan et al., 2021). There have been few studies of m^6^A modification in necroptosis; consequently, the specific regulatory mechanisms involved remain unclear.

## Conclusion and Future Perspectives

Collectively, these findings suggest that m^6^A is essential for regulating PCD. The promotion or inhibition of PCD by m^6^A mainly depends on the level of m^6^A (dynamically regulated by writers and erasers), the function of downstream targets, and the changes in target RNA after methylation (depending on readers). Although some progress has been made in identifying the regulatory mechanisms involved, further research is still needed to explore the exact link between m^6^A modification and PCD in various pathological conditions.

First of all, the current studies mainly focus on the regulatory factors of m^6^A, and the direct influence mechanisms of m^6^A on downstream targets are still unclear. For example, in the study of cell death caused by high glucose, METTL3 mediates pyroptosis by regulating NLRP3 (Zha et al., 2020). Whether METTL3 can directly affect the expression of NLRP3 without an m^6^A-dependent manner and how the changes of m^6^A regulates NLRP3, it still needs further research. Secondly, the regulation of m^6^A is affected by many factors. As mentioned in this paper, METTL3 promotes the installation of m^6^A and FTO inhibits the installation. However, studies have shown that METTL3 up-regulates downstream targets to promote apoptosis, and FTO down-regulates downstream targets to promote apoptosis (Niu et al., 2019; Wang et al., 2020b). This seems to indicate the dual role of m^6^A in apoptosis, which provides a direction for future cancer treatment. Finally, the most challenging issue is that there are often multiple forms of cell death in the same disease. m^6^A may act a “double-edged sword”. For example, m^6^A can not only inhibit the occurrence of cancer by promoting apoptosis and ferroptosis, but also promote the occurrence of cancer by inhibiting apoptosis, pyroptosis, and autophagy. The regulation of m^6^A in the various stages of cell death and the intricate connections among them still need to be further explored.

m^6^A is extremely important for mRNA metabolism at different stages. Except for m^6^A, other chemical modifications also are irreplaceable, such as m^1^A and m^5^C. What is the regulation mechanism of these chemical modifications on PCD? Is it synergistic with m^6^A? These issues still need to be resolved. In general, understanding the link between m^6^A and PCD will not only promote the understanding of the underlying mechanisms of certain diseases, but may also discover new therapeutic strategies.

## Supplementary Material

Supplementary Table S1 The regulatory pathways of different forms of PCD.

Supplementary Table S2 The classification and function of enzymes involved in m^6^A.

Supplementary Table S3 The function of m^6^A in mRNA and ncRNA.

Supplementary Table S4 The roles of m^6^A by regulating apoptosis in the occurrence of cancer.

## Abbreviations

ALKBH, AlkB homolog; ATG, autophagy-related gene; circRNA, circular RNA; EIF3, eukaryotic initiation factor 3; FOXO_3_, Forkhead box O_3_; FTO, fat mass and obesity-associated protein; IGF2BPs, insulin-like growth factor 2; IRF-1, interferon regulatory factor-1; lncRNA, long non-coding RNA; m^6^A, N^6^–methyladenosine; METTL, methyltransferase-like protein; miRNA, microRNA; MTC, methyltransferase complex; mTOR, mammalian target of rapamycin; ncRNA, non-coding RNA; NLRP3, NOD-like receptor protein 3; PCD, programmed cell death; RBM15, RNA binding motif protein 15; SAM, S-adenosylmethionine; SRSF, splicing of arginine-rich splicing factor; TFEB, transcription factor EB; TNF, Tumor necrosis factor; TOLLR, Toll-like receptor; ULK1, uncoordinated 51-like kinase 1; VIRMA, Vir-like m^6^A methyltransferase; WTAP, WT1 associated protein; ZC3H13, zinc finger CCCH-type contains 13.
