# Supplementary material for: m6A: An Emerging Role in Programmed Cell Death
Source: Front Cell Dev Biol. 2022 Jan 24;10:817112. doi: 10.3389/fcell.2022.817112 (PMC8819724; doi:10.3389/fcell.2022.817112)
Supplement: Supplementary file 1 [file Table1.DOCX]

| Types of cell death | Activators | Signal recognizer | Mediators | Target | Outcome |
| --- | --- | --- | --- | --- | --- |
| Autophagy (1-3)  Ferroptosis (4, 5)  Pyroptosis (6, 7)  Apoptosis (8, 9)  Necroptosis (10, 11) | inflammatory factors  Fe^2+^，ROS  inflammatory factors  DNA damage, lack of growth factors  inflammatory factors | AKT/MAPK  AMK/P53  System Xc^-^  NLRP3  Bcl-2  TNFR  TOLLR  TNFR | mTOR  GPX4  Caspase-1  Caspase-11/4/5  Caspase-7/3/8  Caspase-8 | ATG  ULK1  Lipid peroxide  GSDMD  ProIL-1β/18  D1E2V3D4-X  RIPK1/3  MLKL | autophagosome formation  increased mitochondria membrane density  cell membrane formation of pores, cytoplasmic spillover  pyknosis and fragmentation of the nucleus  rupture of cell membranes, spilled intracellular components |
