# Supplementary material for: m6A: An Emerging Role in Programmed Cell Death
Source: Front Cell Dev Biol. 2022 Jan 24;10:817112. doi: 10.3389/fcell.2022.817112 (PMC8819724; doi:10.3389/fcell.2022.817112)
Supplement: Supplementary file 2 [file Table2.DOCX]

| Classification | Gene | Function |  | |
| --- | --- | --- | --- | --- |
| Writers (28)  Erasers (28)  Readers (29-31) | METTL3/METTL14/WTAP/VIRMA/KIAA1429/METTL16  FTO/ALKBH5/ALKBH3  YTH domain/ IGF2BPs /EIF3 | Form MTC to decatalyze the process for m^6^A methylation modification  Mediate m^6^A demethylation modification as demethylases  Recognize the information of m^6^A demethylation modification and participate in downstream RNA translation, degradation and other processes | |  |
