# Supplementary material for: m6A: An Emerging Role in Programmed Cell Death
Source: Front Cell Dev Biol. 2022 Jan 24;10:817112. doi: 10.3389/fcell.2022.817112 (PMC8819724; doi:10.3389/fcell.2022.817112)
Supplement: Supplementary file 3 [file Table3.DOCX]

| Classification | Biological process | Enzyme | | Role of m^6^A | References |
| --- | --- | --- | --- | --- | --- |
| mRNA  ncRNA  miRNA  circRNA  lncRNA | Processing  Export  Translation  Decay  Mature  Translation  Degradation  Stability | FTO/METTL3/YTHDC1  METTL3/ALKBH5  METTL3/EIF3/YTHDF1  YTHDF2  METTL3/METTL14  YTHDF3  YTHDF2  HNRNP/METTL3 | Promotion  Promotion  Promotion  Promotion  Promotion  Promotion  Promotion  Promotion | | (56, 45)  (20)  (43, 48)  (43)  (58, 60)  (53)  (61)  (62, 63) |
