# Supplementary material for: m6A: An Emerging Role in Programmed Cell Death
Source: Front Cell Dev Biol. 2022 Jan 24;10:817112. doi: 10.3389/fcell.2022.817112 (PMC8819724; doi:10.3389/fcell.2022.817112)
Supplement: Supplementary file 4 [file Table4.DOCX]

| Cancer type | Enzyme | Target RNA | Effect of enzyme on target RNA | Role of enzyme on apoptosis in cancer progression | References |
| --- | --- | --- | --- | --- | --- |
| Ovarian cancer  Breast cancer  Lung cancer  Gastrointestinal cancer  Pancreatic cancer  Liver cancer  Renal cell carcinoma  Leukemia  Osteosarcoma | METTL3  YTHDF2  MRTTL3  FTO  METTL14  METTL3  FTO  METTL3  FTO  YTHDF1  FTO  METTL3  FTO  YTHDF2  ALKBH5 | PTEN  -  BCL2  BNIP3  CXCR4  BCL2  MZF1  MEC6  MYC  AKT  PGC-1α  BCL2  MYC  TNFR  YAP | Expression  -  Expression  Expression  Expression  Translation  Stability  Expression  Stability  Expression  Stability  Translation  Expression  Expression  Expression | Inhibits apoptosis to promote the progression  Inhibits apoptosis to promote the progression  Inhibits apoptosis to promote the progression  Promotes apoptosis to inhibit the progression  Inhibits apoptosis to promote the progression  Inhibits apoptosis to promote the progression  Promotes apoptosis to inhibit the progression  Inhibits apoptosis to promote the progression  Promotes apoptosis to inhibit the progression  Inhibits apoptosis to promote the progression  Promotes apoptosis to inhibit the progression  Inhibits apoptosis to promote the progression  Promotes apoptosis to inhibit the progression  Inhibits apoptosis to promote the progression  Promotes apoptosis to inhibit the progression | (99)  (106)  (98)  (92)  (102)  (97)  (93)  (100)  (96)  (105)  (95)  (101)  (94)  (106)  (104) |
